# Employing Solid Phase Microextraction as Extraction Tool for Pesticide Residues in Traditional Medicinal Plants

**DOI:** 10.1155/2016/2890219

**Published:** 2016-09-20

**Authors:** Thamani T. Gondo, Veronica C. Obuseng, Lesego C. Mmualefe, Harriet Okatch

**Affiliations:** ^1^Chemistry Department, University of Botswana, Gaborone, Botswana; ^2^Botswana Institute for Technology Research and Innovation, Gaborone, Botswana; ^3^Perelman School of Medicine, University of Pennsylvania, Philadelphia, PA 19104, USA

## Abstract

HS-SPME was optimised using blank plant sample for analysis of organochlorine pesticides (OCPs) of varying polarities in selected medicinal plants obtained from northern part of Botswana, where OCPs such as DDT and endosulfan have been historically applied to control disease carrying vectors (mosquitos and tsetse fly). The optimised SPME parameters were used to isolate analytes from root samples of five medicinal plants obtained from Maun and Kasane, Botswana. The final analytes determination was done with a gas chromatograph equipped with GC-ECD and analyte was confirmed using electron ionisation mass spectrometer (GC-MS). Dieldrin was the only pesticide detected and confirmed with MS in the* Terminalia sericea *sample obtained from Kasane. The method was validated and the analyte recoveries ranged from 69.58 ± 7.20 to 113 ± 15.44%, with RSDs ranging from 1.19 to 17.97%. The method indicated good linearity (*R* ^2^ > 0.9900) in the range of 2 to 100 ng g^−1^. The method also proved to be sensitive with low limits of detection (LODs) ranging from 0.48 ± 0.16 to 1.50 ± 0.50 ng g^−1^. It can be concluded that SPME was successfully utilized as a sampling and extraction tool for pesticides of diverse polarities in root samples of medicinal plants.

## 1. Introduction

The World Health Organization (WHO) has estimated that 80% of the global population relies on traditional medicine and about 51% of all the drug preparations in industrialized countries are derived from plant or synthesized based on plants extracts [1]. The recent increase in the use of medicinal plants may be due to the fact that they are cost effective as they are ubiquitous in nature when compared to pharmaceutical drugs. For example, Botswana is endowed with a large diversity of plants species which are claimed to have medicinal properties. These medicinal plants are sold in towns and research has indicated that street vendors use around 47 species of plants (distributed in 45 genera belonging to 29 families) to treat various diseases such as skin sores, sexually transmitted diseases, and asthma [2].

However, the safety and efficacy of using some of these medicinal plants have become a major issue [3, 4]. There are various sources of contaminants of unprocessed medicinal plants, such as toxic metals, microorganisms and microbial toxins, radioactivity, fumigation agents, and pesticides [5, 6]. Research on the safety of medicinal plants consumed by local people in Botswana is still limited. For example, Okatch et al. [7] assessed heavy metals content of some medicinal plants from Okavango Delta Region and found that the levels of heavy metals (arsenic, chromium, lead, and nickel) were below the WHO permissible levels. However, there is limited data on the identification and quantification of pesticide residues in medicinal plants used in Botswana.

Organochlorine pesticides such as DDT (dichlorodiphenyltrichloroethane) and endosulfan have been sprayed in the Okavango Delta in an attempt to control disease carrying vectors such as mosquitoes, from as early as the 1940s until the 1990s [8], when the less persistent pyrethroids such as deltamethrin and cypermethrin were introduced. Organochlorine pesticides have been banned (since mid-1970) in most countries due to their persistence in the environment, long range transport, bioaccumulation, and mammalian toxicity [9–12]. Many OCPs have been linked with a broad range of adverse human health and effects, including impaired reproduction, endocrine disruption, and immune-suppression, and they are also considered as carcinogenic substances [13]. Some of these OCPs (e.g., HCB (hexachlorobenzene), aldrin, endosulfan, and DDT and its metabolites) have been detected around Okavango Delta in different matrices such as water [14], sediments [15], and edible plants [16]. Pesticide residues in medicinal plants need to be determined to ensure that they do not exceed maximum recommended levels (MRLs).

Plants present complex matrices that may render trace analysis of pesticide residues quite challenging. Thus good extraction and highly sensitive detection techniques are required to isolate and quantify pesticides in these matrices. Sample preparation on the other hand is prone to errors and consumes about 80% of the analysis time [17]. Traditional extraction methods such as Soxhlet and liquid-liquid extraction have recently received less attention as they are lengthy and require multiple steps resulting in loss of analytes and errors in analysis [17, 18]. For these reasons, recent trends in sample preparation are geared towards miniaturization, automation, high-throughput performance, on-line coupling with analytical instruments, and cost-effectiveness through extremely low or no solvent consumption [19]. Recent techniques for extraction and concentration of pesticides in various plants parts include solid phase extraction (SPE) [20]; quick, easy, cheap, effective, rugged, and safe (QuEChERS) [21]; solid phase microextraction (SPME) [16]; supercritical fluid extraction (SFE) [22]; matrix solid phase dispersion (MSPD) [23]; and pressurized liquid extraction (PLE) [24].

SPME has been utilized for extraction of OCPs from solid matrices because of its ability to preconcentrate analytes; it is solventless and applicable for both volatile and semivolatile analytes, as well as polar and nonpolar compounds [25]. The device affords minimal sample preparation steps accompanied by preconcentration, leading to increased sensitivity [26]. Matrices to which SPME has been employed include water [14, 27–31], soil [32–34], plants materials [16], milk [32], vegetables [35–37], fruits [38, 39], medicinal plants infusions [40, 41], and tea infusions [42–44]. In this work, HS-SPME was applied as a sampling and extraction tool for selected pesticides of varying polarity from selected medicinal plant parts (*Pterocarpus angolensis, Maerua angolensis, Terminalia sericea, Cassia abbreviata, *and* Gymnosporia senegalensis*) obtained from the Okavango Region, Botswana. The objectives of this study are to (i) optimize HS-SPME as a sample clean-up technique of solid samples of medicinal plants and develop a GC-ECD method for determination of organochlorines pesticides in plants and (ii) to apply the optimized method to determine pesticides in medicinal plants from Maun (Okavango Delta) and Kasane.

## 2. Experimental

### 2.1. Material

HPLC grade acetone (99.8%) was obtained from Chromasolv, Sigma-Aldrich (Steinhem, Germany). Pesticide standards, 4,4-DDT (1,1,1-trichloro-2,2-bis[p-chlorophenyl]ethane), *β*-endosulfan, *α*-endosulfan, 4,4-DDE (1,1-dichloro-2,2-bis[p-chlorophenyl]ethylene), aldrin, 4,4-DDD (1,1-dichloro-2,2-bis[p-chlorophenyl]ethane), o,p-DDT (1,1,1-trichloro-2-(o-chlorophenyl)-2-(p-chlorophenyl) ethane), o,p-DDE (1,1-dichloro-2-(o-chlorophenyl)-2-(p-chlorophenyl)ethylene), hexachlorobenzene (HCB), dieldrin, heptachlor epoxide, *α*-hexachlorocyclohexane (*α*-HCH), *β*-hexachlorocyclohexane (*β*-HCH), endrin, PCB 52 (2,2,5,5-tetrachlorobiphenyl; internal standard 1), and PCB 153 (2,4,5,2,4,5-hexachlorobiphenyl; internal standard 2), were all obtained from Sigma-Aldrich, Germany.

15 mL SPME sampling vials, the sampling stand, SPME fiber 65 *μ*m divinylbenzene/polydimethylsiloxane (DVB/PDMS), and fiber holders were all purchased from Supelco (Bellefonte, PA, USA).

Pesticide stock solutions (1000 *μ*g L^−1^) were prepared by dissolving the individual pesticides in acetone. These stock solutions were stored at 4°C (for a maximum period of 3 months) and were used for the preparation of working standard mixtures.

### 2.2. Chromatographic Analysis

#### 2.2.1. GC-ECD

Separation of organochlorine pesticides was achieved using a 7820A gas chromatograph (Agilent Technologies, Durban, South Africa). The system was equipped with a split/splitless injector and ^63^Ni electron capture detector (ECD). An HP-5 MS (5% phenyl methyl siloxane), fused silica capillary column 30 m × 320 *μ*m × 0.25 *μ*m (film thickness) manufactured by J&W Scientific (Torrence, CA, USA) was employed in the separation of analytes. Ultrahigh purity nitrogen gas (99.999%) was used as a carrier gas at a column head pressure of 20.114 Kpa producing a flow rate of 0.5 mL/min. The injector and detector temperatures were set at 250°C and 300°C, respectively. The oven temperature was programmed from an initial value of 70°C (held for 1 min), ramped to 160°C at a rate of 40°C/min (held for 0.5 min), ramped to 180°C at a rate of 35°C/min (held for 1 min), ramped to 240°C at a rate of 4°C/min (held for 2 min), and ultimately ramped to 270°C at a rate of 5°C/min (held for 2 min). The injection volume was 2 *μ*L in the splitless mode.

#### 2.2.2. GC-MS

Confirmation of the pesticides was performed on a 5975C gas chromatography mass selective detector (GC/MSD) system (Agilent Technologies, Santa Clara, USA), with triple-axis high energy dynode-electron multiplier (HED-EM) detector (Agilent Technologies, USA). The GC column and conditions were the same as for GC-ECD analyses as described in the previous section. Helium was used as the carrier gas at a flow rate of 0.5 mL/min. The injector and ion source temperatures were kept at 230°C while the transfer line was kept at 280°C. Full scan (*m/z* 50–600) GC/MS acquisition was carried out at 1 scan sec^−1^. The detector was operated in the electron ionization (EI) mode (70 eV), with an ionization filament emission current of 400 *μ*A. A solvent delay of 4 min was set for the MS to avoid damaging the filament. The mass spectra obtained were compared to the NIST/EPA/NIH Mass Spectral Library, Version 2.0 (Gaithersburg, MD, USA) through Automated Mass Spectral Deconvolution and Identification System (AMDIS) developed by the National Institute for Standards and Technology (NIST) Spectral Library Search. The identities of compounds were also confirmed by comparing their mass spectra with the spectra of the standards and spectra in the NIIST Library and further confirmation (for isomers) was based on comparing the retention times with those obtained for the respective standards.

### 2.3. Sampling and Sample Pretreatment

**Figure 1:**
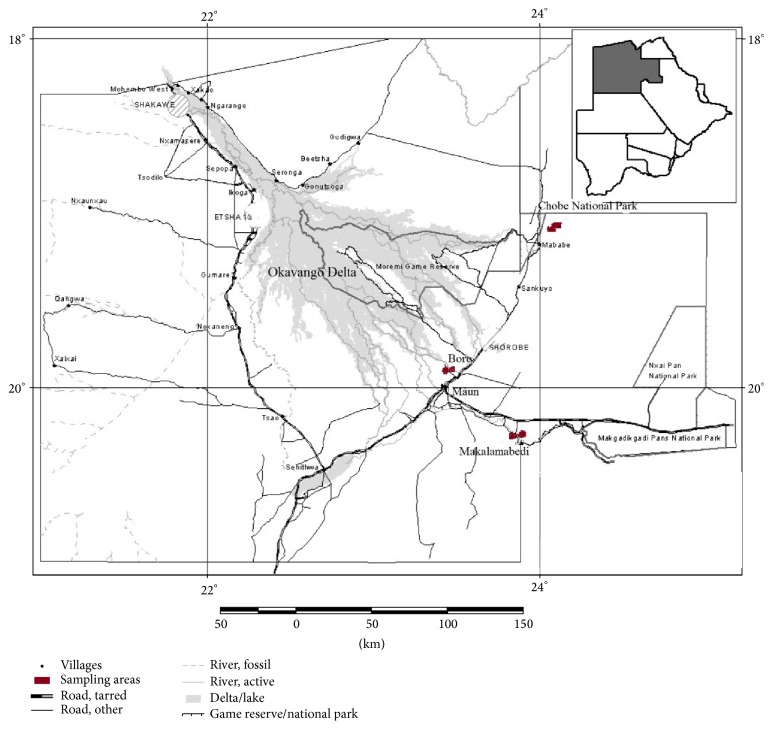
Map of Okavango Delta (Maun) and Kasane areas in Botswana showing the sampling sites.

Medicinal plant specimens (roots parts) were collected in the month of July 2012 from Ngamiland District in the northern part of Botswana (Figure 1). Samples were collected from three different sites, which are as follows: Makalamabedi, situated 56 km south of Maun, along the Boro River, and around Chobe National Park which is situated near Kasane. Medicinal plants were selected based on the interview with traditional healers in the northern part of Botswana who gave information on medicinal plants and the parts used, preparation of remedies, and diseases treated. The plants were collected randomly as the team surveyed and identified the plants in the study areas. The collected samples were wrapped in aluminium foil and then placed in sealed plastic bags stored in ice prior to transporting. The five selected medicinal plants are* Pterocarpus angolensis, Maerua angolensis, Terminalia sericea, Cassia abbreviata, *and* Gymnosporia senegalensis*. The samples were obtained from Okavango Delta except* Pterocarpus angolensis* and another sample of* Terminalia sericea*, which was obtained from Kasane (Chobe). Two* Terminalia sericea* species were obtained from different locations and both plants were analysed to compare the levels of pesticides in those areas. The species were labelled as* Terminalia sericea* A (Okavango Delta, Maun; Figure 1) and* Terminalia sericea* B (Kasane, Chobe). Plant roots samples were dried at room temperature for 24 hours and then cut into small pieces with a clean knife. Dried roots samples were coarsely ground using a pestle and mortar and passed through a 500 *μ*m sieve. These were kept in a dark cold room (4°C) and analysed within a period of four months.

### 2.4. Optimisation of SPME Parameters

A 65 *μ*m PDMS/DVD (medium polarity) was chosen as it is capable of extracting relatively nonpolar to midpolar OCPs [14, 16, 33]. The SPME fiber was conditioned by inserting the fiber into the GC injection port for 1 hour at the 250°C and oven temperature of 200°C while the electron capture detector temperature was set at 300°C. The fiber was inserted into another GC injection port for a further 4 min at 250°C after each and every run as a way of cleaning the fiber to minimise errors as a result of run-to-run carryovers.

*(i) Extraction Temperature.* Optimum extraction temperature was determined by varying temperature between 50 and 100°C for 30 min. 2.5 g selected blank solid plant samples which were collected in area without any history of pesticides use (dry and prescreened for pesticide residues) were weighed and placed in a 15 mL vial, and 125 *μ*L of the standard mixture was added to the sample, shaken for 5 min, and then further kept in the dark for 2 hours. This was followed by the addition of 2.5 mL of water. The 65 *μ*m PDMS/DVB fiber was exposed to headspace to allow adsorption of analytes onto the fiber. 

*(ii) Extraction Time*. Extraction time was determined by weighing 2.5 g dried blank solid plant sample in a 15 mL vial, followed by addition of 125 *μ*L of the standard pesticide mixture before shaking for 5 min that was then further kept in the dark for 2 hours and then adding 2.5 mL of water. The 65 *μ*m PDMS/DVB fiber was inserted into the vial and exposed to the headspace. The extraction procedure was repeated at 10-minute interval between 20 and 50 min. 

*(iii) Phase Ratio*. Phase ratio was investigated by increasing the mass of the homogenised blank sample from 5 to 10 g (i.e., 5, 7.5, 10, and 11.5 g). Each mass was spiked with a concentration of 50 ng g^−1^ by using a spike level of 1000 *μ*g L^−1^ standard mixture and then kept in the dark for 2 hours. The analytes were adsorbed onto the 65 *μ*m PDMS/DVB fiber for 40 min at 90°C and then desorbed onto the GC column. 

*(iv) Volume of Water (Homogenisation)*. The amount of water added to the dry blank plant sample was also optimised by adding different amounts of water ranging from 1.25, 2.5, 5, and 7.5 mL to 2.5 g of dried plant sample after adding 125 *μ*L of 1000 *μ*g L^−1^ standard mixture. The sample was shaken for 5 min and vial was heated to 90°C and then a 65 *μ*m PDMS/DVB fiber was exposed to the headspace for 40 min and then desorbed at 250°C on the injection port. 

*(v) Desorption Time*. 125 *μ*L of 1000 *μ*g L^−1^ standard mixture and 5 mL of water were added to 2.5 g of dried blank plant sample to optimise desorption time at three different times (3, 5, and 7 min).

During optimisation, desorption temperature was kept at 250°C in each case and each study was done in triplicate.

### 2.5. Analytical Parameters

Analytical parameters such as limit of detection (LOD), limit of quantification (LOQ), linearity, accuracy (recoveries), and precision (repeatability) were studied.

Linearity was investigated using a 5-point matrix matched calibration, which ranged from 2 to 100 ng g^−1^. LOD and LOQ were calculated based on signal to noise ratio (S/N) of 3 and 10, respectively, after spiking seven matrix blanks with low concentration of standards (2 ng g^−1^). Recoveries were accessed over 4 concentrations (7, 25, 40, and 70 ng g^−1^) on blank matrices which were found to be pesticide free or undetectable levels of pesticides. The above four concentrations were measured in four replicates and precision was determined in terms of relative standard deviations (RSDs). The precision was also monitored by running a 50 ng g^−1^ spiked blank sample in seven different days (within a week) to check the variation between different days (interday precision). A 50 ng g^−1^ spiked blank was also run for seven times (1 day) to determine the intraday precision. Two internal standards of PCB 52 and PCB 153 were utilised to reduce variations during quantification.

### 2.6. Application of the Developed Method on Real Samples

Pesticides were quantified in five selected medicinal plants used by traditional doctors. Quantification was done using matrix matched calibration curve to compensate for matrix effect. The optimised SPME conditions were used for extraction of pesticides in the five medicinal plants, following analysis by GC.

## 3. Results and Discussion

### 3.1. Optimisation of SPME Conditions

#### 3.1.1. Effect of Extraction Temperature

**Figure 2:**
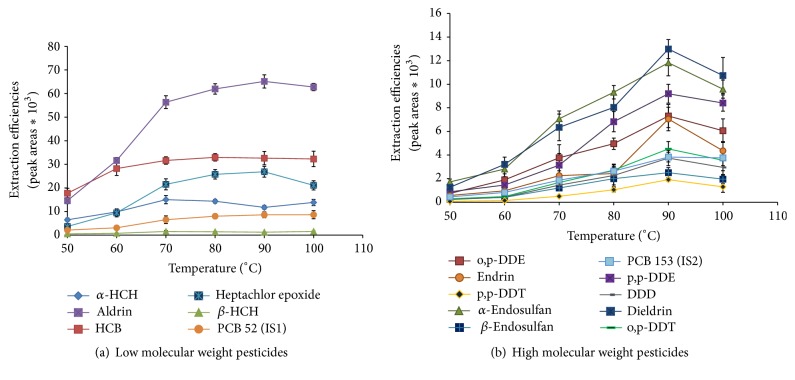
Extraction efficiencies of 14 selected organochlorine pesticides from 50 ng g^−1^ spiked plant root samples, at various temperatures.

Effect of extraction temperature was studied in the range of 50 to 100°C. Generally an increase in temperature resulted in increased diffusion of analytes from the sample to the headspace due to the volatility of the analytes (Figures 2(a) and 2(b)). It was also observed that extraction efficiencies increased with temperature up to 90°C and eventually decreased at temperatures of 100°C for compounds with molecular weight ranging from 318 to 406 g mol^−1^ (aldrin, dieldrin, endrin, heptachlor epoxide, *α*-endosulfan, and *β*-endosulfan; p,p-DDT, p,p-DDE, p,p-DDD, o,p-DDT, and o,p-DDE; Figure 2(b)), except for HCB, *α*-HCH, and *β*-HCH as shown in Figure 2(a). The decrease in extraction efficiencies at higher temperatures might be due to the exothermic nature of the fiber as observed by Doong and Liao [33]. Moreover, higher temperatures increase the solubility of the target analytes in water, hence reducing their distribution coefficients and resulting in decreased extraction efficiencies. In general, compounds with lower molecular weights such as HCB (284.8 g mol^−1^, 323–326°C), *α*-HCH (290.8 g mol^−1^, 288°C), and *β*-HCH (290.8 g mol^−1^, 288°C) enter their gas phase more easily than compounds with higher molecular weights. Analysis of variance (ANOVA) was employed to ascertain the difference in extraction efficiencies at temperatures 70 to 100°C and the results indicated no statistically significant differences in the extraction efficiencies (*F*_calc_ < *F*_critical_, at 95% confidence level). A temperature of 90°C was selected as the optimum extraction temperature for every pesticide since most pesticides indicated high sensitivity at this temperature.

#### 3.1.2. Effect of Extraction Time

**Figure 3:**
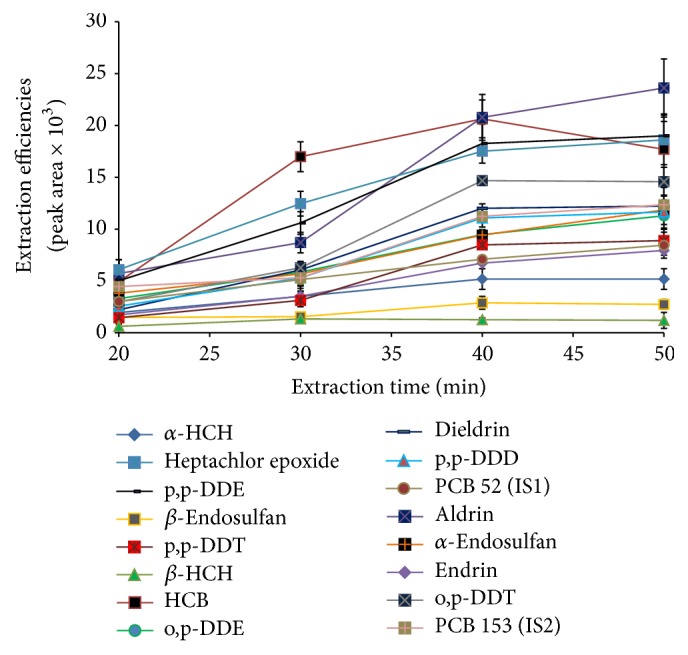
Effect of extraction time on extraction efficiencies (peak areas) of analytes determined using a 50 ng g^−1^ spiked root sample.

In SPME, equilibrium is reached when the analytes adsorbed by the fiber coating are independent of further increase in extraction time. The time required for reaching equilibrium between the fiber and the vapour phase was determined.  Figure 3 shows that most of the analysed pesticides reached the highest extraction efficiencies at 40 min. *β*-HCH was extracted efficiently within a shorter period of time (30 min) due to its low boiling point and partition coefficient (Log *K*_ow_; 3.78). However, one isomer of lindane (i.e., *α*-HCH) did not indicate a significant change from 30 to 50 min as the graph was almost level in that region (this was also proved by ANOVA test). All lindane isomers have relatively low values of Log *K*_ow_ and they exhibit the shortest equilibrium time. HCB indicated a significant drop in extraction efficiencies after an extraction time of 40 min. This could possibly be due to its high volatility resulting in its loss. The graph was almost level after 40 min for all other pesticides in Figure 3 (except HCB and aldrin). It could be that possibly the fiber had reached its maximum loading capacity; thus, the amount of analytes adsorbed was independent of increase in time. Despite some compounds showing extraction times higher than 40 min (i.e., aldrin, heptachlor epoxide, *α*-endosulfan, p,p-DDD, and endrin), the *t*-test (*P* value = 0.05, 95% confidence level) indicated that there was no statistically significant difference; therefore, 40 min was chosen as the optimum extraction time for all the analytes. It should also be noted that a shorter extraction time is crucial in analysis to increase sample throughput.

#### 3.1.3. Phase Ratio

**Figure 4:**
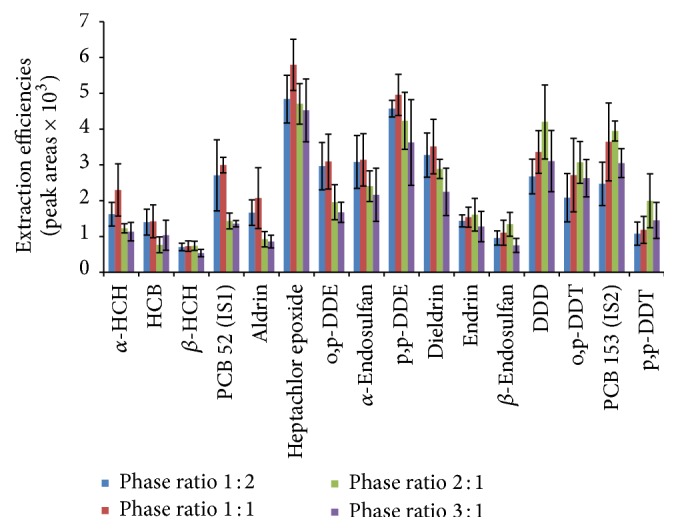
Effect of phase ratio on the extraction efficiencies of analytes.

Phase ratio in this work is defined as the ratio of mass of the solid sample to the volume of the headspace in the vial. The phase ratio was altered by increasing the mass of the homogenized sample in a 15 mL vial, thus altering the headspace volume. Many pesticides showed a drop in extraction efficiencies after a phase ratio of 1 : 1 m/v, whereas the extraction efficiencies of endrin, *β*-endosulfan, p,p-DDD, o,p-DDT, and p,p-DDT reduced at a phase ratio of 3 : 1 m/v (Figure 4). These compounds have higher molecular masses and lower vapour pressure, ranging from magnitudes of 10^−6^ to 10^−7^ mmHg. Their low vapour pressures imply that they do not vaporize easily; hence a high phase ratio is needed to increase their concentration in the headspace. On the other hand, compounds which gave maximum response at a phase ratio 1 : 1 (as shown in Figure 4) have lower molecular masses and higher vapour pressure in the range of 10^−4^ to 10^−5^ mmHg. The low response at lower phase ratio could be due to insufficient equilibration time since in this case a significant portion of the analytes has to be transported to the fiber from the sample and also mass transfer of analytes into the bulk of the fiber represents a slow step in the overall process [45]. A phase ratio of 1 : 1 m/v was selected for this study since most pesticides gave better extraction efficiencies at this ratio.

#### 3.1.4. Sample Homogenisation

**Figure 5:**
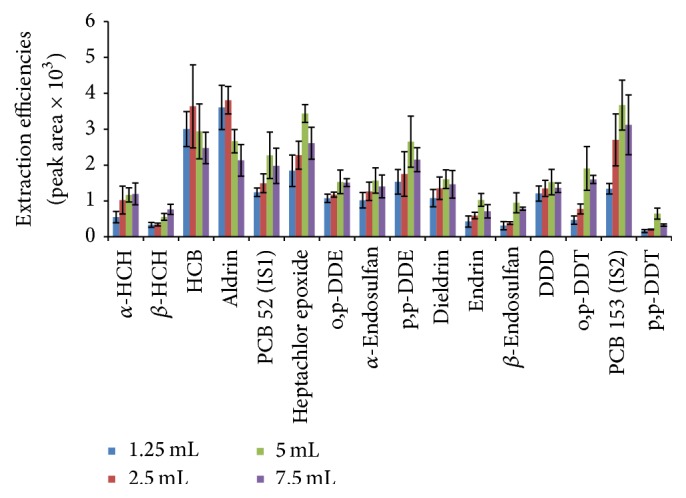
Effect of amount of water added to sample on extraction efficiencies of organochlorine pesticides.

Solid samples are ground into finer particles and water is added to facilitate analyte transfer from the sample matrix into the headspace when performing HS-SPME experiments [33]. This also promotes repeatability in measurements [44]. Water has the ability to open pores in the matrix and also to increase the surface area to enable release of analytes from the matrix.  Figure 5 shows that extraction efficiencies for most pesticides increased when the water volume was increased in the sample up to 5 mL followed by a decrease as the water content was increased.

All these compounds (i.e., heptachlor epoxide, o,p-DDE, *α*-endosulfan, p,p-DDE, dieldrin, endrin, *β*-endosulfan, p,p-DDD, o,p-DDT, and p,p-DDT) are characterized by low water solubility and their vapour pressures are quite lower than those of lindane compounds. However, HCB and aldrin indicate a decrease in extraction efficiencies after water amount was increased to 5 mL. These compounds have low water solubility when compared to *α*-HCH and *β*-HCH and are also characterized by high vapour pressure compared to all compounds which indicated drop in extraction efficiencies at 7.5 mL. It is therefore assumed that increasing the water amount will easily limit their diffusion to the fiber due to increased water vapours in the headspace. It has been reported that relative humidity of 90% can reduce the adsorption by about 10% [46]. The extraction efficiencies of the two isomers of lindane (*α*-HCH and *β*-HCH) increased as water was increased up to 7.5 mL. Lindane compounds are known to be more soluble in water, with low partition coefficients (Log *K*_ow_ is 3.80 and 3.78 for *α*-HCH and *β*-HCH, resp.). The increase in extraction efficiencies might be caused by the type of fiber used, that is, 65 *μ*m PDMS-DVB fiber, since these analytes are partitioned more readily in this fiber as compared to other compounds. Doong and Liao [33] reported a significant increase in extraction efficiencies with a 65 *μ*m PDMS-DVB fiber for extraction of HCH compounds from the soil. Water soluble compounds are easily released from the matrix into water, making these analytes more available for extraction [46]. Furthermore, the high vapour pressure of *α*-HCH and *β*-HCH causes them to be more partitioned into the headspace than in the aqueous solution. However, too much water is detrimental to analyte extraction into the headspace. In this work, 5 mL was selected as the optimum since many compounds indicated high efficiencies at that level.

#### 3.1.5. Desorption Time

**Figure 6:**
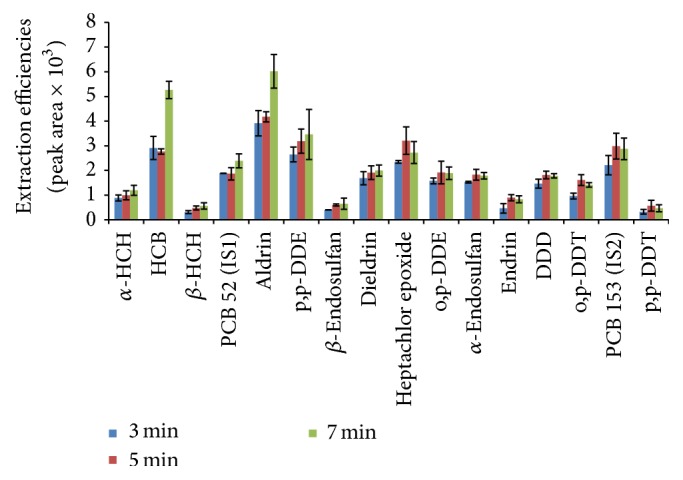
Effect of desorption time of 14 organochlorine pesticides on their extraction efficiencies.

Desorption time influences the number of molecules desorbed into the injector port. Longer desorption time will completely desorb the analytes, but it is also avoided since it can damage the fiber. An increase in extraction efficiencies was observed as desorption time was varied between 3, 5, and 7 min. Desorption time can be influenced by several factors such as the thickness of the fiber, the boiling point of the analyte, desorption temperature, and the partition constant of the analytes. The analytes heptachlor epoxide, o,p-DDE, *α*-endosulfan, endrin, o,p-DDT, p,p-DDD, and p,p-DDT indicated an increase from 3 to 5 min and a drop in extraction efficiencies at 7 min (Figure 6). The drop in extraction efficiencies might indicate complete desorption of particular analytes. Only *α*-HCH, HCB, *β*-HCB, aldrin, p,p-DDE, *β*-endosulfan, and dieldrin indicated slightly higher peak areas at 7 min desorption time (Figure 6). However, the more volatile compounds such as *α*-HCH, HCB, *β*-HCB, and aldrin were expected to give less desorption time, but the reverse occurred. It was also discovered that early eluting compounds (*α*-HCH, HCB, *β*-HCB, and aldrin) are coextracted with some volatile matrix components, which could result in increase in their peak areas, leading to errors. The increase in extraction efficiencies of p,p-DDE might be due to degradation of p,p-DDT on a hot GC inlet surface which is similar to what was observed by other authors [47]. Longer desorption time for *β*-endosulfan and dieldrin might be due to their high molecular weights (relatively high boiling points) and low vapour pressure. On carrying out* t*-test statistics (*P* = 0.05, 95% confidence level), it shows that there was no statistical significant difference between 5 min and 7 min for every pesticide under study. Nevertheless, no significant difference was observed between 3 and 5 min for most pesticides, except for heptachlor epoxide, endrin, and o,p-DDT. A desorption time of 5 min was selected for this study since there was no significant increase in extraction efficiencies after 5 min.

### 3.2. Performance of the SPME Method

Validation of SPME-GC-ECD was applied on spiked blanks which were previously screened and no pesticide was detected.

#### 3.2.1. Recoveries (Accuracy)

Average recoveries were found to range from 69.58 ± 7.20% (*β*-endosulfan) to 113.92 ± 15.44% (*β*-HCH) as shown in Table 1. Low recoveries were observed at lower analyte concentrations for each of the analytes; for example, for 7 ng g^−1^, the lowest recoveries were 69.58 ± 7.20% and for concentrations of 25 ng g^−1^ and 40 ng g^−1^ the lowest recoveries were found to range from 89.66 ± 7.54 to 86.46 ± 3.95%. Most compounds indicated improvement in recoveries at higher concentrations; 70 ng g^−1^ showed recoveries range of 89.59 ± 10.21 to 113.92 ± 15.44%. *β*-HCH indicated the highest recoveries at high concentration (70 ng g^−1^) when compared to all the OCPs. The results might be influenced by the selectivity of the SPME fiber used. The PDMS-DVB fiber has been found to be selective to aromatic hydrocarbons and some small volatile analytes [33], hence high recoveries for *β*-HCH and *α*-HCH. However, most OCPs indicated high recoveries when PDMS-DVB was used, that is, ≥70%; and the fiber also showed a good affinity to the compounds containing phenyl groups such as DDT, DDE, p,p-DDD, and HCB. It was noted that some analytes showed recoveries above 100%. We cannot be absolute on the causes of that; however, these can be due to matrix effects as solid samples are generally difficult to homogenise (especially after spiking), which might result in nonequilibration between the three phases, hence the use of matrix matched standards. These errors are negligible since they were all lower than 20%. In addition the EPA allows recoveries to range from 70 to 120% for a method, thus taking into consideration the analytical errors.

#### 3.2.2. Method Precision

The ranges of RSD were found to be from 5.64 to 17.97%, 5.31 to 13.02%, 3.43 to 12.97%, and 1.19 to 13.04% for concentrations of 7 ng g^−1^, 25 ng g^−1^, 40 ng g^−1^, and 70 ng g^−1^, respectively (Table 1). The intra- and interday precision calculated using a 5 ng g^−1^ spiked standard were found to range from 5.41 to 11.87% and from 5.76 to 15.79%, respectively. It can be noted that better precision was obtained for higher concentrations compared to the lower concentrations. In general, the RSDs were lower than 20% in all cases, indicating low variability between measurements. This result was comparable with other studies; for instance, repeatability ranging from 3.2 to 11.3% was reported for analysis of OCPs in textiles using SPME [48]. In another study, low RSDs were obtained for analysis of OCPs in water, ranging from 5.2 to 14.0% [14]. Fidalgo-Used and coworkers reported repeatability in terms of RSD, in the range of 6 to 28% when SPME was used for OCPs analysis in fish samples [49]. In another study, repeatability for analysis of organochlorines in the soil using SPME was reported to range from 3.2 to 26.3% [33]. Schurek and coworkers [44] also analysed a wide range of pesticides in tea using SPME and obtained repeatability in the range of 2 to 24%, in terms of RSD. Therefore, results were in agreement with other authors' findings.

#### 3.2.3. Linearity

The linearity of the method was tested with matrix matched standards spiked at different concentrations, ranging from 2 to 100 ng g^−1^ (i.e., 2, 10, 25, 50, and 100 ng g^−1^). Good linearity was found for all the analytes with correlation coefficients (*R* ^2^) greater than 0.99 when matrix matched standards were used for calibration. The results are shown in Table 2. The observations are in agreement with the results by other researchers when extracting OCPs using SPME from different matrices, for example, cotton samples [48] and water samples [30, 50], which all indicated good *R* ^2^ (i.e., greater than 0.99 for most analytes).

#### 3.2.4. Detection Limits

The LODs (limits of detection) and LOQs (limits of quantification) were investigated and the results are shown in Table 2. It should be noted that the detection limits determined here were method detection limits (MDLs), which can be regarded as LODs not instrument detection limits (IDLs). The LODs ranged from 0.48 to 1.50 ng g^−1^ after SPME analysis, with a 65 *μ*m PDMS/DVB fiber, whilst the LOQs ranged from 1.61 to 4.80 ng g^−1^. The results were in agreement with results found by Obuseng and coworkers, who indicated detection limits to be ranging from 0.266 to 1.693 *μ*g L^−1^ when SPME was used for the determination of 7 OCPs (i.e., aldrin, endosulfan, p,p-DDD, DDE, dieldrin, endrin, and p,p-DDT) in* Nymphaea nouchali *roots [16]. However, some researchers achieved lower detection limits with SPME method; for instance, in fish tissues low detection limits ranging from 0.1 to 0.7 ng g^−1^ were obtained using SPME and GC-ECD [49]. Low detection limits (0.06 to 0.65 ng g^−1^) were also found when determining 18 OCPs in soil using SPME [33]. Detection limits may vary depending on several factors such as type of the matrix, fiber type, instrument type, and condition. Pérez-Trujillo [51] found different LODs for the same compound when using different SPME fibers in extraction of OCPs. The results indicated that LODs varied from 0.6 to 10.2 ng L^−1^ for the carbowax-divinylbenzene (CW-DVB) fiber, from 0.5 to 11.6 ng L^−1^ for the carboxen-polydimethylsiloxane (CAR-PDMS) fiber, and from 0.4 to 7.4 ng L^−1^ for the divinylbenzene-carboxen-polydimethylsiloxane (DVB-CAR-PDMS) fiber [51]. Most OCPs have indicated low detection limits with 100 *μ*m PDMS fiber, which is sensitive for nonpolar compounds such as o,p-DDE, *α*-endosulfan, p,p-DDE, dieldrin, endrin, *β*-endosulfan, p,p-DDD, o,p-DDT, and p,p-DDT [33, 48, 52].

#### 3.2.5. Analysis of Real Samples

**Figure 7:**
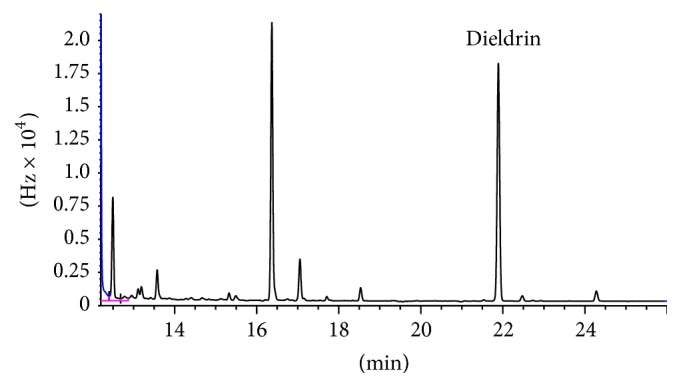
*Terminalia sericea* (B) after HS-SPME-GC-ECD analysis.

SPME optimal conditions (i.e., extraction temperature, 90°C; extraction time, 40 min; phase ratio, 1 : 1; volume of water, 5 mL; and desorption time, 5 min) were applied to the roots of real samples, which are* Pterocarpus angolensis, Maerua angolensis, Terminalia sericea, Cassia abbreviata*, and* Gymnosporia senegalensis*. Only dieldrin was confirmed with MS and its concentration was found to be 150.5 ± 8.4 ng g^−1^ in* Terminalia sericea* B with ECD (Figure 7). However, some low levels of pesticides were detected with ECD in other plants, but these results could not be reliably confirmed since MS could not confirm the peaks due to high detection limits for the compounds of interest in MS as compared to ECD.

In Botswana, dieldrin was used in 1964 [53, 54] to control tsetse fly and mosquitoes in the areas of Okavango Delta and Kasane. Dieldrin has not been documented in any case from previous studies in Botswana. Since more studies about pesticides have been focused on the region of Okavango Delta, there is a need to look into the region of Kasane since pesticides were also applied in that region. Studies have also shown that aldrin can readily undergo oxidation to its more persistent epoxide, dieldrin [55]. The World Health Organization established the acceptable daily intake (ADI) of dieldrin as 100 ng kg^−1^ body weight and the oral reference dose (RfD) as 50 ng kg^−1^ day^−1^ [56]. The EU maximum residue levels (MRLs) in milk are reported to be 6 ng g^−1^ [32], which is much less than what was detected in the sample. The high levels of dieldrin detected in* Terminalia sericea* B can possibly be explained by its persistence in the environment. Estimated half-life of dieldrin in the environment has been reported to be up to 25 years [56, 57].

## 4. Conclusion

Solid phase microextraction method for the analysis of OCPs in solid plants samples was successfully developed. HS-SPME combined with GC-ECD has been shown to be simple, fast (less steps), cheap, solventless, reproducible, and effective for the analysis of OCPs in medicinal plants. High recoveries in the range of 69.58 ± 7.20 to 113.92 ± 15.44% were attained. Optimisation of parameters also yielded low LODs (lower than 2 ng g^−1^), high precision of the method, and good linearity (*R* ^2^ ≥ 0.99). LODs ranged from 0.48 to 1.50 ng g^−1^, and the RSDs were found to be below 20% for all pesticides. Only dieldrin was detected in the roots of* Terminalia sericea* species obtained from Kasane; however, the quality of medicinal plants in terms of pesticides was satisfactory since none were detected in most of them.

## Supplementary Material

Method precision was evaluated by injecting a 50 ng/g spiked blank sample and measuring the repeatability within a day and also within a week. The first data set labelled runs represents repeatability performed in one day and done in replicate and the second data set represents repeatability measured in different days in one week. SD represents standard deviation and RSD represents relative standard deviation (%).

## Figures and Tables

**Table 1:** % recoveries and their standard deviations (mean ± SD) after analysis of spiked blank samples. The precision of the method calculated as % RSD for four measurements of each sample.

	Mean recoveries							
	7 ng g^−1^	% RSD	25 ng g^−1^	RSD	40 ng g^−1^	RSD	70 ng g^−1^	RSD
*α*-HCH	90.87 ± 6.26	6.89	91.46 ± 8.06	8.82	92.07 ± 10.72	11.64	94.47 ± 3.16	3.28
HCB	83.79 ± 9.01	10.76	81.54 ± 10.48	12.86	86.46 ± 3.95	4.56	89.59 ± 10.21	12.11
*β*-HCH	76.62 ± 8.05	10.50	81.90 ± 9.57	11.69	87.03 ± 11.29	12.97	113.92 ± 15.44	12.82
Aldrin	85.36 ± 10.96	12.84	91.46 ± 5.46	5.97	91.56 ± 4.48	4.89	95.35 ± 6.32	6.14
Heptachlor	81.82 ± 3.76	4.60	99.28 ± 13.33	13.43	98.70 ± 5.81	5.89	101.77 ± 4.72	4.69
o,p-DDE	74.76 ± 6.50	8.69	88.78 ± 9.01	10.15	101.94 ± 5.96	5.85	102.03 ± 5.74	5.52
*α*-Endosulfan	75.97 ± 11.96	15.74	103.21 ± 4.71	4.56	105.01 ± 11.43	10.89	107.36 ± 6.66	6.55
p,p-DDE	75.80 ± 8.78	11.24	97.98 ± 12.76	13.02	98.39 ± 3.37	3.43	105.96 ± 2.56	2.41
Dieldrin	75.04 ± 8.41	11.58	93.01 ± 9.88	10.63	97.60 ± 9.88	10.12	107.25 ± 11.90	11.68
Endrin	93.43 ± 5.27	5.64	95.68 ± 8.22	8.59	106.34 ± 5.38	5.06	107.07 ± 5.56	5.53
*β*-Endosulfan	69.58 ± 7.20	10.35	95.76 ± 10.04	10.49	110.59 ± 5.58	4.86	112.74 ± 1.34	1.19
p,p-DDD	82.78 ± 8.87	10.72	98.45 ± 5.26	5.35	99.44 ± 8.06	8.11	102.56 ± 8.23	8.02
o,p-DDT	103.71 ± 8.62	8.31	100.40 ± 11.27	11.23	101.04 ± 6.55	6.48	102.95 ± 5.54	4.90
p,p-DDT	76.39 ± 13.73	17.97	89.66 ± 7.54	8.41	98.84 ± 5.57	5.64	98.36 ± 12.82	13.03

**Table 2:** Linearity, LODs, and LOQs of OCPS after SPME procedure.

	Equation	Correlation coefficient	LOD (ng g^−1^)	LOQ (ng g^−1^)
*α*-HCH	*y* = 0.168 −0.7642	0.9906	1.00 ± 0.33	3.35 ± 0.33
HCB	*y* = 0.0866 + 0.3576	0.9985	1.44 ± 0.48	4.80 ± 0.48
*β*-HCH	*y* = 0.0111 − 0.0155	0.9933	1.50 ± 0.50	4.99 ± 0.50
Aldrin	*y* = 0.1025 − 0.1005	0.9971	0.49 ± 0.16	1.62 ± 0.16
Heptachlor epoxide	*y* = 0.06 − 0.1873	0.9970	0.73 ± 0.24	2.43 ± 0.24
o,p-DDE	*y* = 0.0147 − 0.0314	0.9978	0.76 ± 0.25	2.53 ± 0.25
*α*-Endosulfan	*y* = 0.0289 − 0.053	0.9967	0.99 ± 0.32	3.30 ± 0.32
p,p-DDE	*y* = 0.0197 − 0.0206	0.9978	1.34 ± 0.45	4.46 ± 0.45
Dieldrin	*y* = 0.0234 − 0.0452	0.9963	1.20 ± 0.40	4.01 ± 0.40
Endrin	*y* = 0.0364 − 0.1672	0.9900	0.60 ± 0.20	2.00 ± 0.20
*β*-Endosulfan	*y* = 0.0087 + 0.011	0.9983	1.32 ± 0.44	4.41 ± 0.44
p,p-DDD	*y* = 0.0105 − 0.0202	0.9971	0.78 ± 0.25	2.59 ± 0.25
o,p-DDT	*y* = 0.0298 − 0.1573	0.9919	0.48 ± 0.16	1.61 ± 0.16
p,p-DDT	*y* = 0.0101 − 0.0232	0.9975	1.19 ± 0.40	3.98 ± 0.40
